# 

**Published:** 1883-01

**Authors:** 


VOL. IV.] TWO DOLLARS A YEAR. SINGLE COPIES 60 CENTS. [No. 1.

### THE JOURNAL OF COMPARATIVE MEDICINE AND SURGERY.

### A QUARTERLY JOURNAL

OF THE

ANATOMY, PATHOLOGY AND THERAPEUTICS OF THE LOWER ANIMALS.

Entered New York Post Office as second class matter.

### JANUARY, 1883—CONTENTS.

WILLIAM R. JENKINS,

VETERINARY PUBLISHER, BOOKSELLER AND PRINTER,

850 Sixth Ave., Cor. 48th Street,

NEW YORK.

